# Introduction to celebrating Latin American talent in chemistry†

**DOI:** 10.1039/d1ra90175c

**Published:** 2021-12-17

**Authors:** Gabriel Merino, María A. Fernández-Herrera, Galo J. A. A. Soler-Illia, Aldo J. G. Zarbin, Vânia G. Zuin, Eduardo Chamorro, Luciana G. de Oliveira, Márcia Foster Mesko, Cesar Fraga, Ilich A. Ibarra Alvarado, Jairton Dupont, Ana Flávia Nogueira, Carlos F. O. Graeff, Heloise Oliveira Pastore, Eufrânio N. da Silva Júnior, Omar Azzaroni

**Affiliations:** Departamento de Física Aplicada, Centro de Investigación y de Estudios Avanzados Unidad Mérida. Km. 6 Antigua Carretera a Progreso, Apdo. Postal 73, Cordemex 97310 Mérida Yuc. Mexico gmerino@cinvestav.mx mfernandez@cinvestav.mx; Instituto de Nanosistemas, UNSAM, CONICET Av. 25 de Mayo 1021, 1650 San Martín Buenos Aires Argentina gsoler-illia@unsam.edu.ar; Departamento de Química, Universidade Federal do Paraná (UFPR), CP 19032, CEP 81531-980 Curitiba-PR Brazil aldozarbin@ufpr.br; Departamento de Química, Universidede Federal de São Carlos, CEP 13565-905 Rod. Washington Luískm 235 – SP-310 São Carlos-SP Brazil; Facultad de Ciencias Exactas, Departamento de Ciencias Químicas, Universidad Andres Bello Avenida República 275 8370146 Santiago Chile echamorro@unab.cl; Departamento de Química Orgânica, Universidade Estadual de Campinas (UNICAMP), CP 6154, CEP 13083-970 Campinas-SP Brazil; Centro de Ciências Químicas, Farmacêuticas e de Alimentos, Universidade Federal de Pelotas Capão do Leão RS Brazil; Laboratório de Avaliação e Síntese de Substâncias Bioativas (LASSBio), Instituto de Ciências Biomédicas, Universidade Federal do Rio de Janeiro PO Box 68023 Rio de Janeiro RJ Brazil; Instituto de Investigaciones en Materiales, Universidad Nacional Autónoma de México Circuito Exterior s/n, CU, Coyoacán 04510 Ciudad de México Mexico; Institute of Chemistry – Universidade Federal do Rio Grande do Sul. Av. Bento Gonçalves 9500 Porto Alegre 91501-970 RS Brazil; University of Campinas (UNICAMP), Laboratório de Nanotecnologia e Energia Solar, Chemistry Institute PO Box 6154 Campinas Brazil; São Paulo State University (UNESP), School of Sciences, Department of Physics Bauru São Paulo Brazil; Institute of Chemistry, University of Campinas Monteiro Lobato St. 270 Campinas São Paulo 13084-971 Brazil; Institute of Exact Sciences, Department of Chemistry, Federal University of Minas Gerais Belo Horizonte Minas Gerais Brazil; Instituto de Investigaciones Fisicoquímicas Teóricas y Aplicadas (INIFTA), Departamento de Química, Facultad de Ciencias Exactas, Universidad Nacional de La Plata (UNLP), CONICET 64 and 113 La Plata (1900) Argentina

## Abstract

In celebration of the excellence and breadth of Latin American research achievements across the chemical sciences, we are delighted to present an introduction to the themed collection, Celebrating Latin American talent in chemistry.
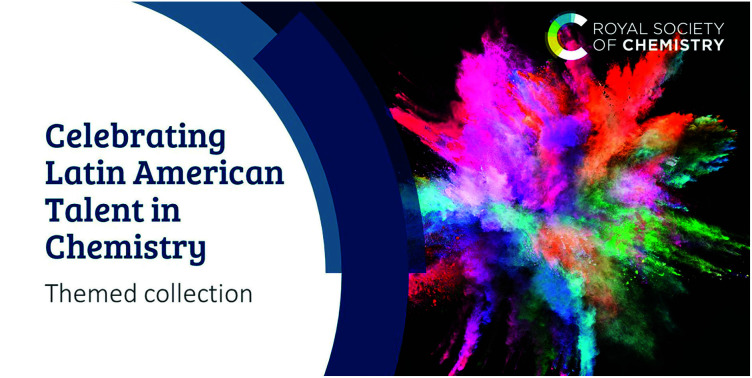

A year ago, amid the pandemic, we proposed that the RSC should compile a collection of Latin American contributions to chemistry. The central aim of this collection is to showcase the quality of the research work being carried out in this area. The project was quite well received, and several journals joined. This is not a unique project in this sense; there have been other recent collections with the purpose of highlighting the chemistry developed in Latin America.^1–5^ However, the current situation of chemistry in each region deserves an analysis of its respective historical context. So, we have taken advantage of this space first to provide a very general historical overview of chemistry in Latin America and then to analyze certain numbers that offer us a picture of where we stand as a consequence of our history. We apologize for focusing on only three countries in a region that includes 46 countries (Latin America and the Caribbean), but we are sure that the stories and problems are similar.

## A very short history of chemistry in Latin America

We do not pretend to give a compendium of the history of Latin American chemistry, but some facts are common to the development of this area in our countries throughout the years.^6,7^ Before the arrival of the European navigators, standardized chemical processes used in paints, foodstuffs, and construction had already been developed. During the colonial period, the exploitation of natural resources led to the generation of new chemical techniques used in different economic cycles, such as mining and the extraction and transformation of natural resources such as rubber and sugar. Indeed, the name Brazil was chosen because of the strong red pigment extracted from the pau-brasil tree (*Paubrasilia echinata Lam.*).

The techniques developed for the mining industry became perhaps the first triumphs of Latin American chemistry. One of the most eminent Brazilian chemists and local political leaders (he is considered the patriarch of independence for his fight for Brazil’s independence from Portugal) of the 18^th^ century was José Bonifácio Andrada e Silva, who discovered and identified twelve different new minerals, among them petalite, which is directly related to the discovery of lithium.^8^ Another example is Andrés Manuel Del Río, who strongly promoted teaching, research, and the extraction of iron ore in several regions. In 1801, he analyzed a mineral known as *brown lead*, extracted from a mine in Hidalgo, and isolated a new element called *Panchromium* (many colors) and later *Erythronium* (red). Convinced of the discovery and lacking the necessary tools to complete his analysis, he sent the ore to Europe to be tested. In Paris, it was concluded that the sample contained only chromium and lead. Thirty years later, Sefström rediscovered it and renamed it vanadium. Although Del Río died without knowing these results, history has given him his place as the discoverer of vanadium.

The independent countries in Latin America continued to be immersed in an economic model based on foreign investment and the export of raw materials (unfortunately, it is still the prevailing paradigm) instead of promoting the country’s industrial and scientific development. Chemistry was initially an auxiliary discipline to medicine, pharmacology, and the food industry. In the mid-19^th^ century, the rise of agricultural industries prompted the first research efforts and the establishment of a professional community dedicated to solving chemical problems. From an academic and professional approach, chemistry began in the early years of the 19^th^ century, for example, with the Department of Industrial Chemistry of the National Medical Institute in Mexico, aimed at obtaining alkaloids and producing active pharmaceutical ingredients extracted from plant and animal species. In Argentina, shortly after independence, in 1821, the University of Buenos Aires was founded, and the first chair of chemistry was established, headed by Manuel Moreno.^9^ A milestone in the separation of chemistry from pharmacy and medicine is the creation in 1896 of the Doctorate in Chemistry at the same institution, later extended to the universities of La Plata and Litoral at the beginning of the 20^th^ Century. The School of Chemical Engineering was created at the Universidad del Litoral, which became a new beacon of applied chemistry. In the meantime, the teaching of chemistry became popular at middle and high school levels. In Mexico, the National School of Industrial Chemistry was created in 1916, becoming a Faculty of Chemistry and Pharmacy in 1919. In Brazil, the first institution entirely dedicated to science was the Scientific Academy of Rio de Janeiro, created in 1772. Afterwards, several colleges and scientific institutions were created in the 19^th^ century, and the first universities were founded at the beginning of the 20^th^ century. Accordingly, the Brazilian Academy of Sciences was created in 1916.

Until the 1930s, Mexico experienced an upward development of chemistry at the academic level. Given its biodiversity, chemistry in Mexico focused on natural products research. The abundant biodiversity attracted U.S. scientists to Mexico to collect plant species rich in hormone precursors. This led, in 1951, to the synthesis of norethindrone, which became the active ingredient of the first contraceptive pill. Together with Carl Djerassi and George Rosenkranz, Luis Miramontes patented this compound. Miramontes’ work on the contraceptive pill is considered Mexico’s most significant contribution to science.^10^ The contraceptive pill is acclaimed worldwide, marking a change as valuable as the wheel or the internal combustion engine.^11^

In 1941, the Institute of Chemistry of the National Autonomous University of Mexico (UNAM) was created. This institute had a laboratory for organic chemistry and natural products, where chemistry students were trained in research, and researchers were given a full-time appointment to dedicate themselves exclusively to this task. Subsequently, several institutes were created to address issues related to petrochemistry and natural products and to develop new research areas, such as the Chemistry departments of Cinvestav and the Universidad Autónoma Metropolitana (UAM), and the Instituto Mexicano del Petróleo (Mexican Petroleum Institute).

The lack of adequate working conditions, insufficient financial support, or simply the fact that certain areas have not been fully developed has provoked a brain drain. Among many examples, Mario Molina stands out, a Mexican educated at UNAM, who did his PhD at the University of California at Berkeley. During his postdoctoral stay with Rowland, Molina became interested in certain industrial chemicals, chlorofluorocarbons (CFCs) and their movements in the atmosphere, discovering that the dissolution of CFCs affected the ozone layer. Years later, he was awarded the Nobel Prize “for his work in atmospheric chemistry, especially with regard to the formation and decomposition of ozone”.

In Argentina, the work of Chemistry Nobel Prize Laureate Luis F. Leloir (1970) and Houssay’s disciples (Houssay Nobel Prize in Physiology, 1947) is an example of a very productive research community in biochemistry that remains a landmark of Argentinian science.^12^ Unfortunately, the promising development of Argentinian science suffered devastating blows in the 1966–1982 period. In July 1966, after a military coup d’état to president Arturo Illia, the *de facto* government decided to intervene at the University of Buenos Aires. Police forces invaded the Schools of Sciences and Philosophy and violently evicted the university authorities. This brutal event, known as “*la noche de los bastones largos*” (so-called because of the long batons with which the police attacked students and professors), caused a massive brain drain. More than a thousand personnel, including more than 300 professors, resigned; many of them emigrated to other Latin American countries (Brazil, Chile and Venezuela), the U.S, Canada, and Europe. One example is the case of César Milstein, an Argentine researcher who won the Nobel Prize in 1984 for his discovery of monoclonal antibodies. Milstein returned to Argentina from Cambridge in 1961. The military coup of 1962 and subsequent racial and political persecution made him decide to continue his career in Cambridge. The advent of a feeble democracy in the 1970s brought some researchers back to the country. However, a precarious political and economic situation, followed by the years of terror between 1976 and 1982, in which many researchers were killed or disappeared, had a significant impact on the community.^13^ From 1966 onwards, the separation of the academy (more radical) and industrial (more conservative) communities became evident, which meant less added value for local products. The advent of democracy in 1983 led to a more stable situation, the scientific community benefited from freedom of thought and speech, and some improvements took place.

One Latin American science success in history, in terms of investment and thus knowledge generation, is Brazil. Part of this success is due to the creation in 1951 of the Coordination for the Improvement of Higher Education Personnel (CAPES) and the National Research Council (now CNPq) governmental bodies in charge of formulating and executing science policy, assessment, and education. Although part of the research in chemistry is carried out in the National Research Institutes, approximately 90% of chemistry in Brazil is associated with the 74 postgraduate programs, primarily in public universities. Chemistry in Brazil experienced significant growth at the beginning of the 21^st^ century due to a continuous increase in the budget dedicated to science since 2004. This budget rose in 2015, 350% more than in 2004. The investment growth was accompanied by an increase in the number of PhD graduates (from 8000 per year to approximately 19 000 per year in the period 2004–2015) and in the number of scientific publications, in which Brazil is currently the 13^th^ country in the ranking of scientific publications,^14^ being responsible for approximately 2% of all scientific papers published in the world in 2020. However, the increasing trend in the budget for Science and Technology reversed after 2015 and experienced a steady decline year after year, reaching in 2021 the lowest budget in the entire history of the sector (only one-third of the total invested ten years ago).

Today, chemical research in Latin America has diversified. Inorganic chemistry, analytical chemistry, organic chemistry, physical chemistry, biochemistry, materials chemistry, chemistry education and computational chemistry have positioned Latin American researchers as leaders within the international scientific community, mainly due to the combination of substantial individual efforts and networking with central countries. The fields of green energies and the environment are growing, and strong clusters have been built in nanotechnology, biotechnology and biorefinery in the last decades. Although some incremental advances and some high-quality hotspots exist, several barriers are reflected in the stagnation of the number of publications and patents relative to global progress. These include a lack of academic–industry connections, an outdated infrastructure, and insufficient highly trained chemists. The current challenges for Latin America involve a reconceptualization of the role of chemistry in society, with particular emphasis on the modernization of the educational curriculum at all levels and the necessary improvement of interactions between academia and industry.

In terms of support, Latin American researchers have been supported for some decades by a reasonable (but far from sufficient and appropriate) budget for stipends and grants, allowing us to generate high-quality knowledge regardless of the differences with the global north. The future, however, is alarming; drastic reductions in research funding are increasing every year, and the scientific community has been undervalued by our governments. We cannot go back two centuries to an economic model based on an incipient extractive and processing industry. Hopefully, the changes taking place globally due to pandemics and climate change will clarify the value of the contribution from the chemistry community in Latin America.

## Some numbers

With the historical background in place, let us focus on some statistics. Although we concentrate on the contributions published in RSC, these figures can be generalized (*vide infra*). As mentioned, the collection was quite well received, and 24 journals were added. Given the number of contributions and space limitations, only manuscripts published during 2019 and 2020 were considered. During these two years, 1451 articles were published from Latin America out of a total of 54 011, or 2.7% (Table 1, ESI).^15^ In these same journals, from 2011 to 2020, 259 768 articles were published, of which 6187 (2.4%) have an author based in Latin America (Table 2, ESI), *i.e.*, this percentage increases a little bit over almost a decade.

When the search is open to all contributions catalogued in chemistry, regardless of editorials and in the same time frame, there are a total of 99 107 (Table 3, ESI) out of 2 492 360 (4.0%, see Table 4, ESI). These almost 100 000 contributions produced 1 379 238 citations or 13.9 citations per article. Now, if we classify these manuscripts according to their subject areas, the main contributors were: general chemistry (32.6%), physical and theoretical chemistry (18.0%), organic chemistry (16.4%), and analytical chemistry (13.6%). The countries with the highest number of contributions are Brazil (53 175), Mexico (19 471), Argentina (12 050), Chile (7349), and Colombia (5645), Table 5, ESI. Although our number of published articles (99 234) as a region is slightly lower than that of the UK (108 609) and higher than that of Spain (84 906), the reality is that the impact, quantified as the number of citations per publication, is lower (Latin America: 13.9, UK: 26.9, Spain: 23.6). This is where our situation is reflected (Table 6, ESI).

The choice of a representative sample of different areas, groups, and countries is *per se* a thorny problem. The selection was carried out (a total of 208), taking into account that the groups were based in Latin America and trying to maintain the balance between areas and countries. For the selection, two criteria were applied: the corresponding author affiliation address must be from a Latin American country, and in cases of corresponding authors with multiple contributions, the most representative publication was considered. We are sure that this blind selection resulted in a fair and representative sample.

## Final reflections

History and numbers make it clear that we must seek mechanisms to urge the recognition of Latin American talent in chemistry, both at home and abroad. It is imperative that our governments invest more resources in research and protect science through adequate public policies to close the existing gaps with other regions of the world. We also need gender equity, a change in curricula, and the strengthening of STEM programs in the region, as well as to promote greener technologies and sustainability in a wider sense. Another important point is to demand a more significant presence of Latin American scientists in editorial boards, scientific societies, and international forums (as @LatinXChem), which will allow us to participate in decision-making, considering a balanced diversity (*e.g.*, gender, age, area of expertise, among others). As Cereijido argued, our countries should abandon the concept of “supporting the development of science” and embrace instead “supporting their development on science”.

## Supplementary Material

RA-011-D1RA90175C-s001
