# Supplementary material for: Introduction to celebrating Latin American talent in chemistry
Source: RSC Adv. 2021 Dec 17;11(63):40216–9. doi: 10.1039/d1ra90175c (PMC9044753; doi:10.1039/d1ra90175c)
Supplement: RA-011-D1RA90175C-s001 [file RA-011-D1RA90175C-s001.pdf]

Electronic Supplementary Material (ESI) for RSC Advances.
This journal is © The Royal Society of Chemistry 2021

Latin America Themed Collection

Gabriel Merino,[1,*] María A. Fernández-Herrera,[1] Galo J. A. A. Soler-Illia,[2] Aldo J. G. Zarbin,[3] and Eduardo Chamorro.[4]

[1]Departamento de Física Aplicada, Centro de Investigación y de Estudios Avanzados, Unidad Mérida. Km. 6 Antigua Carretera a Progreso, Apdo. Postal 73, Cordemex, 97310, Mérida, Yuc., México.

[2]Instituto de Nanosistemas, UNSAM, CONICET, Av. 25 de Mayo 1021, 1650 San Martín, Buenos Aires, Argentina.

[3]Departamento de Química, Universidade Federal do Paraná (UFPR), CP 19032, CEP 81531-980 Curitiba-PR, Brazil.

[4]Facultad de Ciencias Exactas, Departamento de Ciencias Químicas, Universidad Andres Bello, Avenida República 275, 8370146 Santiago, Chile.

E-mail: gmerino@cinvestav.mx

Table 1-SI. Number of publications indexed in Web of Science published in a set of RSC journals within the period 2019-2020. (Data exported: 17-Oct-2021).

**Journal**	**Number of publications**	**Number of publications from Latam**
Analyst	1678	37
Analytical Methods	1315	97
Chemical Communications	6447	63
Chemical Science	2743	30
Chemical Society Reviews	487	12
CrystEngComm	1705	43
Dalton Transactions	3757	122
Energy Environmental Science	625	8
Environmental Science Nano	590	11
Environmental Science Processes Impacts	356	4
Environmental Science Water Research Technology	470	16
Food Function	1624	78
Green Chemistry	1446	43
Journal of Materials Chemistry A	5094	60
Journal of Materials Chemistry B	1670	24
Journal of Materials Chemistry C	3420	40
Materials Advances	344	15
Natural Product Reports	144	8
Organic Biomolecular Chemistry	2186	59
Physical Chemistry Chemical Physics	5437	296
RSC Advances	9423	293
RSC Medicinal Chemistry	120	10
Soft Matter	2013	64
Sustainable Energy Fuels	917	18
**Total**	**54011**	**1451**

Table 2-SI. Number of publications indexed in Web of Science published in a set of RSC journals within the period 2011-2020. (Data exported: 17-Oct-2021).

**Journal**	**Number of publications**	**Number of publications from LatAm**
Analyst	7874	199
Analytical Methods	8407	617
Chemical Communications	32669	313
Chemical Science	8490	93
Chemical Society Reviews	3369	36
CrystEngComm	10384	188
Dalton Transactions	19013	443
Energy Environmental Science	3651	39
Environmental Science Nano	1287	22
Environmental Science Processes Impacts	1504	40
Environmental Science Water Research Technology	945	21
Food Function	4318	263
Green Chemistry	5750	129
Journal of Materials Chemistry A	19713	172
Journal of Materials Chemistry B	6697	104
Journal of Materials Chemistry C	11349	144
Materials Advances	344	15
Natural Product Reports	708	24
Organic Biomolecular Chemistry	11477	272
Physical Chemistry Chemical Physics	28370	1288
RSC Advances	61038	1457
RSC Medicinal Chemistry	120	10
Soft Matter	10894	270
Sustainable Energy Fuels	1397	28
**Total**	**259768**	**6187**

Table 3-SI. Overall research performance for publications in chemistry generated from Latin America within the period 2011-2020. Data source: Scopus (Scival). Data exported: 20 October, 2021.

Indicator	Value
Scholarly output	99107
Citation count	1379238
Citations per publication	139
**Publications by Subject Area**	
General Chemistry	41836
Physical and Theoretical Chemistry	22997
Organic Chemistry	21000
Analytical Chemistry	17392
Spectroscopy	8710
Inorganic Chemistry	8158
Electrochemistry	4945
Chemistry (Miscellaneous)	2968

Table 4-SI. Overall research performance for publications in chemistry generated in the World within the period 2011-2020. Data source: Scopus (Scival). Data exported: 20 October, 2021.

**Indicator**	**Value**
Scholarly output	2492360
Citation count	48490177
Citations per publication	19.5
**Publications by Subject Area**	
General Chemistry	1143455
Physical and Theoretical Chemistry	533534
Organic Chemistry	503629
Analytical Chemistry	311986
Spectroscopy	188422
Inorganic Chemistry	222423
Electrochemistry	121831
Chemistry (Miscellaneous)	47966

Table 5-SI. Number of publications in the chemistry area generated from Latin America countries within the period 2011-2020.

Latin America Country	Scholarly output
Brazil	53176
Mexico	19492
Argentina	12049
Chile	7357
Colombia	5741
Venezuela	1286
Cuba	1234
Uruguay	1085
Ecuador	973
Peru	659
Puerto Rico	642
Costa Rica	408
Panama	191
Bolivia	100
Paraguay	66
Dominican Republic	46
El Salvador	33
Guatemala	28
French Guiana	26
Honduras	23
Nicaragua	20
Guadeloupe	12
Martinique	6
Haiti	4

Table 6-SI. Benchmark multiple metrics for publications in the chemistry area from China, US, UK, Spain and Latin America within the period 2011-2020. Data source: Scopus (Scival). Data exported: 20 October, 2021.

**Country**	**Scholarly output**	**Citations per publication**
China	712738	21.1
United States	395423	29.0
United Kingdom	108609	26.9
Latin America	99234	13.9
Spain	84906	23.6
